# High-Performance Resistance-Switchable Multilayers of Graphene Oxide Blended with 1,3,4-Oxadiazole Acceptor Nanocomposite

**DOI:** 10.3390/mi10020140

**Published:** 2019-02-20

**Authors:** Lei Li, Guangming Li

**Affiliations:** 1 Key Laboratory of Functional Inorganic Material Chemistry (MOE), School of Chemistry and Materials Science, Heilongjiang University, Harbin 150080, China; lileidtk@hlju.edu.cn; 2 HLJ Province Key Laboratories of Senior-Education for Electronic Engineering, Heilongjiang University, Harbin 150080, China

**Keywords:** PBD:GO nanocomposites, multilayers of GO charge-trap, rewritable memristor, fluorescent quencher GO

## Abstract

Graphene oxide (GO) has been actively utilized in nonvolatile resistive switching random access memory (ReRAM) devices due to solution-processability, accessibility for highly scalable device fabrication for transistor-based memory, and cross-bar memory arrays. Uncontrollable oxygen functional groups of GO, however, restrict its application. To obtain stable memory performance, 2-tert-butylphenyl-5-biphenyl-1,3,4-oxadiazole (PBD) a that can serve as 1,3,4-oxadiazole acceptor was carefully introduced onto the GO framework. Better stability was achieved by increasing the weight ratio of the chemical component from 2:1 to 10:1 in all GO-based solutions. Particularly, rewritable nonvolatile memory characteristics were dependent on the ratio between PBD and GO. PBD:GO devices with a proportion of 10:1 w/w exhibited better memory performance, possessed a higher ON/OFF ratio (>10^2^) at a lower switching voltage of −0.67 V, and had a long retention ability. The interaction between PBD and GO can be demonstrated by transmission electron microscope, scanning electron microscope, thermogravimetric analysis, fourier transform infrared spectra, Raman spectra, X-ray diffraction, and fluorescence spectra. The superior ReRAM properties of the multilayers of GO blended with the PBD nanocomposite are attributed to electron traps caused by the strong electron acceptors.

## 1. Introduction

In modern von Neumann computer systems, all the fetching, decoding, and execution of instructions are dependent on binary algorithms. Any physical phenomena that generate bistable states, such as magnetism, ferroelectricity, and phase transition, can be utilized to develop random-access memories [1,2,3,4,5]. Among the emerging memory technologies, resistive random-access memory (ReRAM), which stores binary digital data by denoting “0” and “1” separately as the low and high conductivity in response to an applied electric field, is of great significance for innovation in electronic industries. ReRAM requires rational design and the synthesis of novel functional materials with desired and controllable electronic performance. Organic memories have been proposed to revolutionize electrical applications by providing extremely inexpensive, lightweight, and transparent modules, which can be fabricated onto plastic, glass, or the top layer of the complementary metal-oxide semiconductor (CMOS) circuits [6,7,8,9,10,11,12]. Flexible and miniaturized memory devices with a simple structure can be manufactured via solution processing, resulting in a simplified fabrication process and a considerably reduced manufacturing cost. In controlling the device’s performance by means of molecular design and chemical synthesis, organic chemistry, more importantly, provides a treasure trove of functional molecules that can adjust the electronic properties of organic materials.

Organic small molecules, as the most promising candidate to realize high-performance electrical data storage, have been widely investigated in organic field-effect transistors, light-emitting diodes, and solar cells [13,14,15]. Their advantageous properties include low cost, a well-defined molecular structure, easy purification, and optoelectrical property tenability. Oxadiazole-containing organic small molecules with a nitrogen-containing heterocyclic aromatic structure are a topic of great interest because of their remarkable properties. The electron-efficient oxadiazole moiety has been employed in the molecule design of organic optoelectronic materials to enhance electron mobility. In electroluminescence devices, incorporating oxadiazole-containing organic materials can greatly enhance the overall device efficiency, balancing the charge mobility in the active organic components. Therefore, bistable memory effects can be predicted due to the strong electron-withdrawing ability of the 1,3,4-oxidiazole group. 2-tert-butylphenyl-5-biphenyl-1,3,4-oxadiazole (PBD) is one of the most efficient hole blocking/electron transporting materials, because of its high electron affinity and wide band gap.

Graphene Oxide (GO) has been actively utilized in nonvolatile ReRAM devices due to its solution-processability, accessibility for highly scalable device fabrication for transistor-based memory, and cross-bar memory arrays. Uncontrollable oxygen functional groups of GO, however, restrict its application. To obtain stable memory performance, we introduced accepting electronic moieties to GO flakes to study the effect of the weight ratio of the chemical component between PBD and GO on electrical memory behaviors. Investigation on a resistance-switchable mechanism was fully characterized by techniques such as transmission electron microscope (TEM), scanning electron microscope (SEM), thermo gravimetric analysis (TGA), FT-IR spectra, Raman spectra, X-ray diffraction (XRD), and fluorescent spectra. The memory devices, which utilizes multilayers of GO blended with a 1,3,4-oxadiazole acceptor, demonstrated bistably rewritable solution-processed ReRAM devices. The superior ReRAM properties of the multi-component-based device are attributed to the electron traps caused by the strong electron acceptors.

## 2. Materials and Methods

### 2.1. Materials

PBD (the formula weight *F*_w_ = 354.44 g/mol) was purchased from Sigma, China-Mainland. For the multilayers of GO (purchased from Hengqiu Tech. Inc., Suzhou, China), the purification was more than 90 wt% while the thickness, diameter together with the number of graphene sheets separately ranged from 3.4 nm to 7 nm, 10 μm to 50 μm, 6 to 10 layers.

### 2.2. Fabrication of Resistive Switching Bearing Multilayers of GO Blended with PBD

A resistive switching memory device, indium tin oxide (ITO)/PBD:GO/Ni, bearing multilayers of GO blended with PBD, was fabricated on an ITO quartz glass substrate. Prior to the fabrication of the organic layers, the substrate was cleaned with organic solvents, acetone, methanol, and ethanol in an ultrasonic bath. The blends were spin-coated onto the ITO/quartz glass from 1-methyl-2-pyrrolidone (NMP) solution (5 mg/mL). The solution, mixed with the 1,3,4-oxidiazole acceptor and GO, was formed by using an ultrasonic treatment at 27 °C for 4 h. Then, the homogeneous GO-mixed PBD (mixing ratio of 2:1, 5:1, 10:1, w/w) solution was coated on the ITO-coated glass substrate at 1000 rpm for 60 s at room temperature. The active layer was subsequently baked at a vacuum ambient for 24 h. The thickness of the PBD:GO nanocomposite layer, as measured by a NanoMap 500LS 3D Profilometer, was approximately 50 nm. Finally, a cathode of Ni (250 nm) was deposited through a shadow mask under a high vacuum (10^−5^ torr), whose active area of the device was 200 μm^2^.

### 2.3. Measurements

The morphology of the samples was measured by a JEM-2100 transmission electron microscope (JOEL, Tokyo, Japan) operating at 200 kV. The cross-section of the nanocomposite films with a small molecule embedded into the GO framework was characterized by an Apreo Scanning Electron Microscope (Themoscientific, Waltham, MA, USA). Moreover, aNanoMap 500LS 3D Profilometer (aep Technology, Santa Clara, CA, USA) was adopted to measure the nanocomposite film thickness. Furthermore, TGA (TA Instruments, New Castle, DE, USA) was used to analyze thermal properties of PBD:GO composites under a nitrogen atmosphere at a heating rate of 10 °C/min. A foss DS 2500 Infrared Spectrometer was employed to test fourier-transform infrared spectroscopy (FTIR) spectra of PBD:GO composites, with a chemical composition proportionality of 2:1, 5:1, and 10:1 (w/w), scanning from 600 nm to 4000 nm. Raman spectroscopy (Horiba Jobin Yvon, Villeneuve-d’Ascq, France) were separately employed to gain structure information of PBD:GO composites with the proportional chemical composition 2:1, 5:1, and 10:1 (w/w), scanning from 100 cm^−1^ to 4000 cm^−1^. XRD (X’Pert PRO XRD meter, Panalytical, Finland) was recorded with Cu Kα radiation (wavelength = 1.540598 Å), for which the scans were taken in the 2*θ* range of 5°–30° with scan step size of 0.0131303°/mm. Fluorescence spectra were measured with a F-4500 FL Spectrophotometer (HITACHI, Tokyo, Japan), in 2.5 nm/2.5 nm slit widths, in response time of 2 s, and at scan speed of 1200 nm/min. Fluorescence is a novel method to investigate, based on a GO charge-trap, the resistance-switchable mechanism of memristic characteristics. High-performance resistive switching was performed with a source meter (Keithley 4200SCS; Keithley, Solon, OH, USA) at 300 K. Memory performance of the devices was all conducted under ambient conditions, without any encapsulation.

## 3. Results

### 3.1. TEM and SEM Images

**Figure 1:**
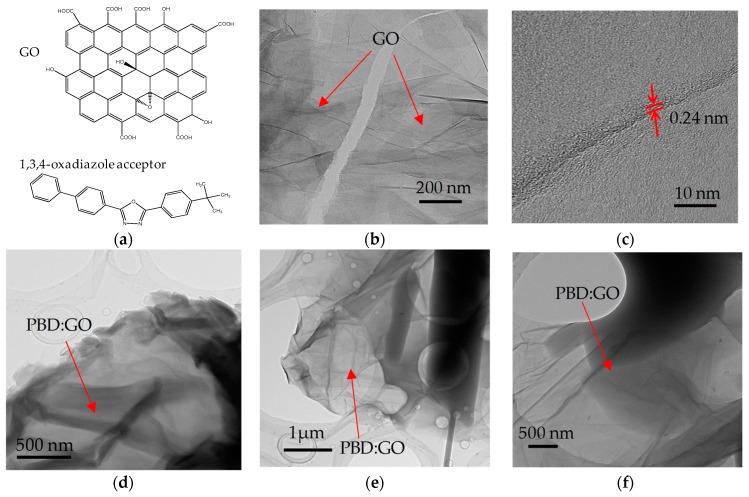
Micrographs of graphene oxide (GO) and 2-tert-butylphenyl-5-biphenyl-1,3,4-oxadiazole (PBD):GO nanocomposites. (**a**) Structure of 1,3,4-oxdiazole acceptor and a schematic representation of multilayers of GO; (**b**) Transmission electron microscope (TEM) and (**c**) High-Solution TEM micrographs of GO sheets; (**d**–**f**) TEM images of PBD:GO nanocomposite with distinct weight ratio 2:1, 5:1, 10:1 (w/w).

Schematic diagrams of the resistive switching bearing a 1,3,4-oxadiazole acceptor embedded in multilayers of GO are illustrated in Figure 1a, and the chemical structure of the GO framework with partially oxidized GO molecules is shown in Figure 1b,c. TEM images of PBD embedded in multilayers of GO, with distinctive chemical proportions of 2:1, 5:1, and 10:1, are separately indicated in Figure 1d–f.

**Figure 2:**
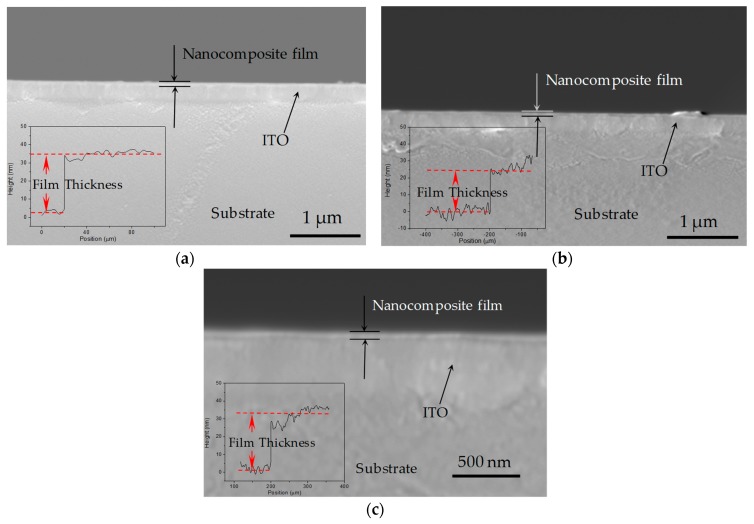
Scanning electron microscope (SEM) images of cross-sectional characterization and film thickness by Profilometer for PBD:GO nanocomposite films with the chemical component of weight ratio (**a**) 2:1, (**b**) 5:1, and (**c**) 10:1. Indium tin oxide, ITO.

PBD is dispersed in multilayers of GO, resulting in the formation of PBD:GO nanocomposite materials. Multilayers of GO are randomly distributed. The PBD distributed in the GO layer is immobilized on the GO surface, which might be attributed to the interaction between PBD and the GO layer with COOH and OH groups. However, the higher the GO content, the more folding-cluster aggregations are observed in the composite, due to the strong p-p tacking within GOs [16]. Figure 2 provides the cross-section of the PBD:GO hybrid films with different weight ratios of 2:1, 5:1, and 10:1, respectively. The thickness of the hybrid films was measured by Profilometer, roughly up to 30 nm.

### 3.2. Memory Behavior Measurments

**Figure 3:**
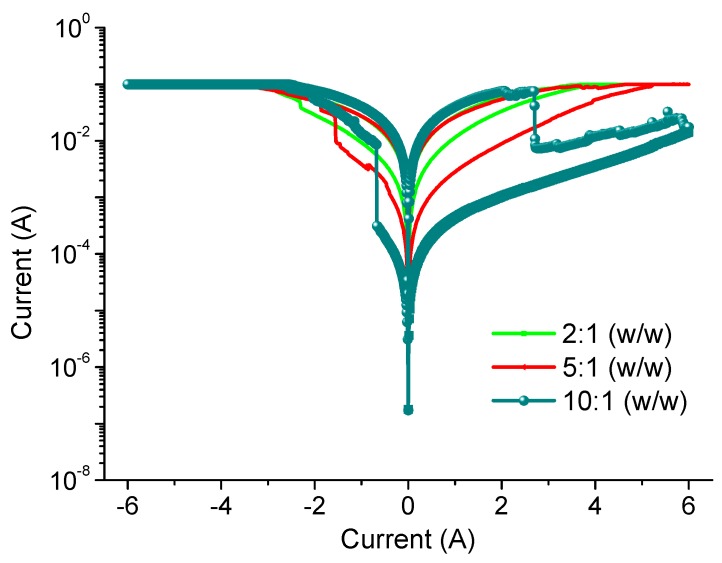
*I*-*V* curves of ITO/PBD:GO/Ni with weight ratio of the composite 2:1, 5:1, and 10:1.

*I*-*V* measurements were performed by two-terminal methods to investigate the charge transport in GO films. The devices with diverse chemical proportions of PBD:GO exhibit similar electrical behaviors when measured under ambient conditions. The devices have the capability for excellent switching performance, allowing the possibility for use in nonvolatile memory. Electrical experiments were conducted with the chemical component PBD:GO, with a weight ratio of 2:1, 5:1, and 10:1 (w/w), as an active layer sandwiched between ITO and Ni electrodes. The device with PBD:GO of 2:1 (w/w) exhibits electrically bistable switching behavior, as illustrated by the *I*-*V* characteristics in Figure 3. In the first voltage scan from 0 V to −6 V, the current grows abruptly at a threshold voltage of about −2.3 V, switching from an OFF-state to an ON-state. The device keeps in ON-state during the subsequent sweep from 0 to −6 V (sweep 2). The distinct electrical states in the voltage range of about 0 V to −6 V, where the ON/OFF current ratio is more than 3.5, allow a voltage of −0.1 V to read the ‘‘0’’ signal (before setting) and ‘‘1’’ signal (after setting) of the memory device. For the subsequent positive voltage scan (sweep 3), an abrupt decrease in the current can be observed at about +3.2 V. The device can be reset to the OFF-state (sweep 4) for a nonvolatile rewritable memory device. Further decreases in GO content result in a significant change in the conductivity of the composite film, and the devices with PBD:GO of 5:1 (w/w) also exhibit rewritable resistive switching behavior. The threshold voltage, when the devices switch from an OFF-state to an ON-state, can reach −1.55 V. Interestingly, the ratio between low- and high-conductive states can be stably obtained up to 11, resulting in a nonvolatile memory device. Furthermore, the rewritable memory device with 10:1 (w/w) of PBD:GO is initially in an OFF-state; when the bias voltage increases, a transition from an OFF-state to an ON-state occurs at a lower threshold voltage of −0.68 V, presumably because of the oxidation of 1,3,4-oxadiazole groups. The ON/OFF current ratio is approximately 130 when read at −0.1 V. A nonvolatile ON state is thus observed. The ON state can be retained after removing the power supply (sweep 2). After reading the ON state in the negative sweep, a positively biased sweep with a sufficient magnitude of −2.71 V (sweep 3) can program the ON state back to the initial OFF state (erase process). The OFF state of the device can be read (sweep 4) and re-programmed to the ON state again in subsequent negative sweeps, thus completing the write-read-erase-read- rewrite cycles for a nonvolatile rewritable memory. The resistive switching observed may be the result of trapping/detrapping of the PBD:GO active layer, which acts as a capacitor. We further investigated the endurance cycles, device uniformity, and retention ability of the devices, which are parameters required for application as a nonvolatile memory device.

**Figure 4:**
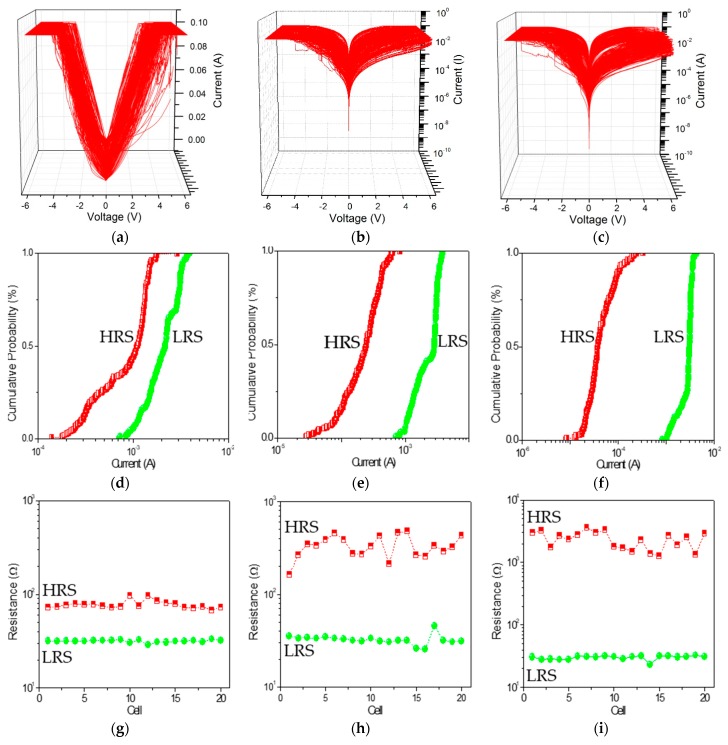
(**a**–**c**) Endurance cycles, (**d**–**f**) statistical analysis, and (**g**–**i**) device-to-device profiles of 20 devices at *V*_read_ = −0.1 V with the chemical component ratio of PBD:GO at 2:1, 5:1, and 10:1 w/w.

**Figure 5:**
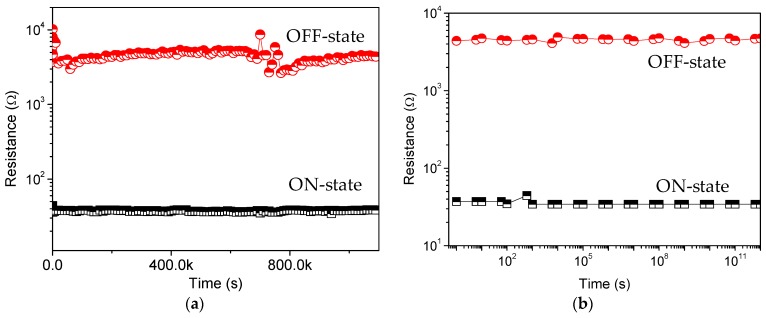
Retention ability of the PBD:GO memory with 10:1 (w/w) at a (**a**) constant and (**b**) pulse of reading voltage 0.1 V.

Nanocomposites can exert an exceptional influence on nano elements in a long-term stability, specifically, to enhance the ON/OFF current ratio of the bistable resistive switching. PBD:GO nanocomposite can be treated as an organic storage material for bistably electrical performance. 200 consecutive cycles of *I*-*V* characteristics for ITO/PBD:GO/Ni were carried out and plotted in Figure 4a–c. The rewritable cycles can be repeatedly performed without significant degradation after several hundred rewritable cycles. The cumulative distribution of *I*_HRS_ and *I*_LRS_ at *V*_read_ = −0.1 V is displayed in Figure 4e,f. The mean (standard deviation) of *I*_HRS_ at a read voltage of −1 V is 0.97 mA (0.49 mA), 0.26 mA (0.15 mA), and 0.55 μA (0.0.46 μA), while that of *I*_LRS_ is 2.20 mA (0.80 μA), 2.40 mA (0.93 μA), and 2.80 mA (0.75 μA). For the resistive switching separately ITO/PBD:GO(2:1)/Ni, ITO/PBD:GO(5:1)/Ni, and ITO/PBD:GO(10:1)/Ni, the device uniformity of 20 electrically bistable samples were considered and calculated. Device-to-device profiles of *I*_HRS_ and *I*_LRS_ at *V*_read_ = −0.1 V are illustrated in Figure 4g–i. The extent of *I*_HRS_ and *I*_LRS_ are signed by the red and green dashed lines. The observations show that the nanocomposite PBD:GO (10:1 w/w) has better memory characteristics. An ON/OFF state resistance ratio *R*_ON_/*R*_OFF_ of more than 10^2^ at −0.1 V has been achieved in the bistable memory device ITO/PBD:GO(10:1)/Ni. No significant degradation of the device in both the ON and OFF states is observed after 10^6^ s of the continuous stress test (Figure 5a), indicating that both the composite materials and the electrode/nanocomposite interfaces are stable. *R*_ON_/*R*_OFF_ in the bistable devices is high enough to promise a low misreading rate through precise control of the ON and OFF states. The effect of continuous read pulses with a read voltage of −0.1 V in the ON and OFF states is also studied. As shown in Figure 5b, more than 10^12^ read cycles are conducted on the ITO/PBD:GO/Ni devices, and no resistance degradation is observed for the ON and OFF states. Neither the voltage stress nor the read pulses cause state transition because the applied voltage (−0.1 V) is lower than the switching threshold voltage. Thus, both states are stable under voltage stress and are insensitive to read pulses. These results confirm that the device has excellent rewritability for nonvolatile memory device applications. Thus, a larger ON/OFF ratio with a low driving voltage is desirable in the field of nano-scaled molecular electronics.

### 3.3. Thermal and Optical Measurements

**Figure 6:**
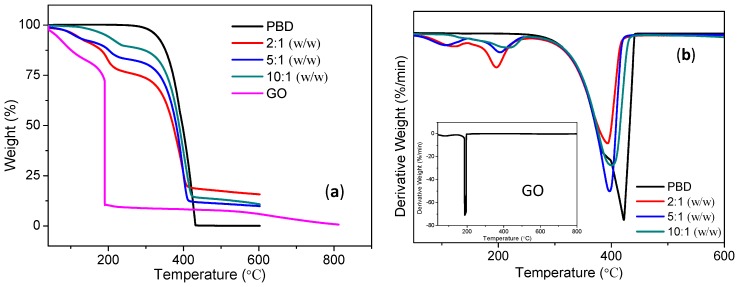
Characterization of GO, PBD:GO nanocomposite, PBD. (**a**) Thermo gravimetric analysis (TGA) and (**b**) differential thermo-gravimetric (DTG) properties of GO (in Inset), PBD:GO nanocomposites with 2:1, 5:1, and 10:1 (w/w), and PBD.

In order to understand the performance and mechanism of the switching behavior of the PBD:GO device, the thermal and optical measurements were also investigated. To determine the thermal stability of the samples, TGA and differential thermo-gravimetric (DTG) of PBD, GO, and PBD:GO composites were carried out under a nitrogen atmosphere at a heating rate of 10 °C/min. The TGA and DTG curves obtained from PBD:GO nanocomposite samples are shown in Figure 6. It can be observed that distinct degradation behaviors occur. In Figure 6a, the decomposition temperature with a weight loss of about 5% for PBD and GO is 327 °C and 60 °C, while that for PBD:GO with 2:1 (w/w), 5:1 (w/w), and 10:1 (w/w) is respectively 112 °C, 109 °C, 193 °C. It can be seen that PBD completely degrades at ~400 °C, while GO has 0.67% weight when the temperature approaches 800 °C. The thermal degradation process for PBD:GO composites has two steps. The DTG curves of the PMMA:GO composites exhibited in Figure 6b have strong peaks at 395 °C, 397 °C, and 401 °C, suggesting that the incorporation of different amounts of GO significantly shifts the temperature of thermal degradation, and the magnitude of the shift is dependent on the amount of GO. Most likely, small molecules that have inhibiting effects on the thermo-oxidative degradation of PBD are adsorbed on the surface of GO.

**Figure 7:**
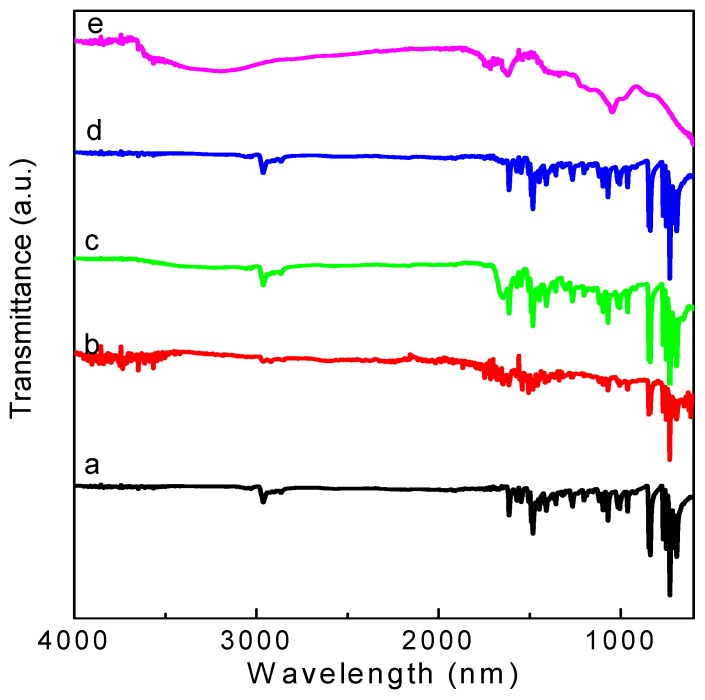
Fourier-transform infrared spectroscopy (FTIR) spectra of (**a**) PBD, (**b**–**d**) PBD:GO composites with chemical components of weight ratio 2:1, 5:1 and 10:1, respectively, (**e**) GO.

**Figure 8:**
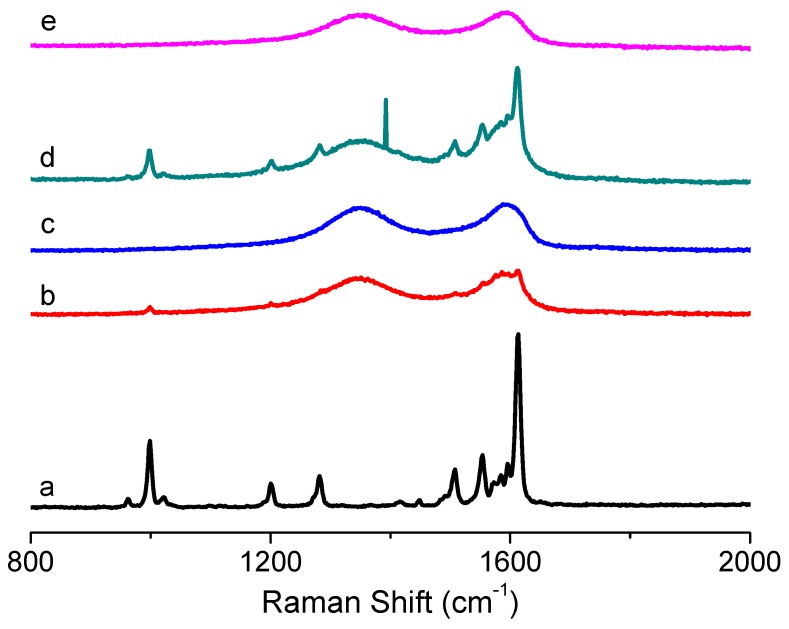
Raman spectra of (**a**) PBD, (**b**–**d**) PBD:GO composites with chemical components of weight ratio 2:1, 5:1 and 10:1 (w/w), respectively, (**e**) GO.

As shown in Figure 7, the main characteristic absorption bands in the FTIR spectra of GO are located at 1732 cm^−1^ (C = O carbonyl stretching of -COOH), 1418 cm^−1^ (O-H deformation vibration), 1171 cm^−1^ (C-OH stretching), and 1047 cm^−1^ (C-O stretching). The broad peaks for GO at 3400 cm^−1^ and 1620 cm^−1^ are derived from the stretching vibration of hydroxyl (–OH) group and the skeletal vibration of the graphene sheets [17], respectively. The above mentioned phenomena present various oxygen functional groups on the GO surface. FTIR spectra for the PBD:GO blends are almost the same as those of PBD. The spectra provide evidence that oxadiazole units were successfully incorporated into the blends. The incorporation of the 1,3,4-oxadiazole acceptor is evidenced by the medium intensity at 1570 cm^−1^ for –C = N– stretching of the oxadiazole ring, as well as the medium-strong band at 1015 cm^−1^ derived from the C–O–C vibration or heteroatom ring deformation of the oxadiazole ring. Moreover, C = C linkages in aromatic rings are responsible for the absorption peaks at 1613 cm^−1^ and 1490 cm^−1^. Figure 8 shows the Raman spectra of PBD:GO compared with those of GO and PBD. For PBD, the obvious peak stemming from the C–O–C stretching mode of the 1,3,4-oxadiazole ring is at 999 cm^−1^. The stronger Raman activity can be found at 1613 cm^−1^ now that it has the C–C stretching pattern in the benzene ring. Under excitation with a 532 nm laser, the Raman spectrum of GO displayed two prominent bands at about 1354 cm^−1^ (D-band) and 1596 cm^−1^ (G-band), with the *I*_D_/*I*_G_ of 0.93. The D-band has usually been used to monitor the process of covalent functionalization, which transforms sp^2^ to sp^3^ sites, while the G-bands can be utilized to estimate the level of chemical modification. The D-band and G-band of PBD:GO with 2:1, 5:1, and 10:1 (w/w) are located at 1355 cm^−1^ and 1595 cm^−1^, 1350 cm^−1^ and 1587 cm^−1^, and 1342 cm^−1^ and 1584 cm^−1^ respectively, with a blue-shift compared to those of GO. This change implies a decrease in the average size of the sp^2^ domains upon reduction of GO. After absorbing the small molecules to the surface of GO, the G-bands were largely enhanced and blue-shifted to 1584 cm^−1^. The *I*_D_/*I*_G_ ratio decreased from 0.93 to 0.67.

**Figure 9:**
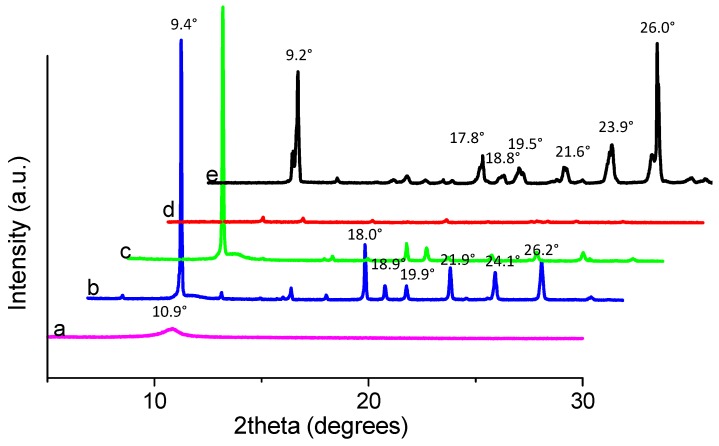
X-ray diffraction (XRD) spectra of (**a**) GO, (**b**–**d**) PBD:GO blends with 2:1, 5:1, 10:1 (w/w), respectively, and (**e**) PBD.

To understand the change in the interlayer of GO films with different chemical ratios, X-ray diffraction (XRD) was used. The XRD patterns of the three compounds are shown in Figure 9. The diffraction peaks of the compounds, compared with those of PBD, occur at larger degrees. For example, PBD shows diffraction peaks at 2*θ* = 9.2° (9.6 Å), while the compounds are at 2*θ* = 9.4° (9.4 Å), which demonstrates that compounds have a closer intermolecular staking distance. In reality, the composite with the chemical component ratio PBD:GO of 10:1 (w/w) has the same peaks as the composites with ratios of 2:1 and 5:1 (w/w), but its intensities are so weak that the diffraction peaks for the composite with PBD:GO of 10:1 (w/w) seem inconspicuous. For PBD:GO blends, the intensity for various peaks is obviously associated with the weight ratio of the chemical component, decreasing with incremental PBD content. The small molecules absorbing to the surface of GO may destroy the crystal structure of the PBD to some extent and reduce its crystallinity degree. The increasing content of PBD is responsible for the intensity reduction. This phenomenon may be due to the interaction between PBD and GO that leads to the change of the crystal structure of the samples [18]. The peak for the (002) facet of graphene at 2*θ* = 10.9° (8.1 Å) is also shown. According to the XRD of PBD, the other peaks arise at 2*θ* = 17.8° (4.98 Å), 18.8° (4.72 Å), 19.5° (4.55 Å), 21.6° (4.11 Å), 23.9° (3.72 Å), and 26° (3.42 Å), while others for the composites correspondingly exhibit at 2*θ* = 18° (4.92 Å), 18.9° (4.69 Å), 19.9° (4.46 Å), 21.9° (4.06 Å), 24.1° (3.69 Å), and 26.2° (3.4 Å). These results indicate that intermolecular π-π stacking, which would facilitate charge transport through the neighboring molecules, became stronger [19].

**Figure 10:**
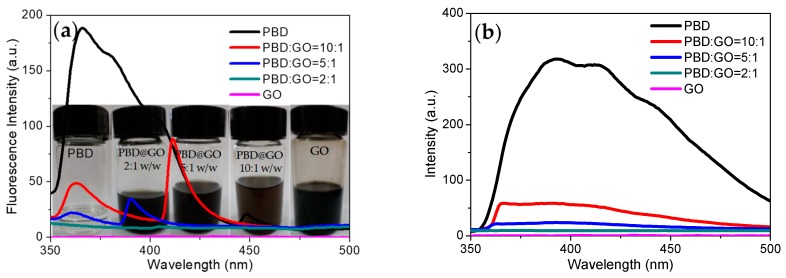
(**a**) Fluorescence excitation spectra and (**b**) fluorescence emission spectra of PBD, PBD:GO blends, and GO solutions at *λ*_em_ = 400 nm and *λ*_exc_ = 360 nm. Photos of PBD, PBD:GO blends, and GO in 1-methyl-2-pyrrolidone (NMP) solutions in Inset.

For the fluorescence studies, fluorescence excitation and emission of these compounds in the NMP solution get readily quenched by GO, with increasing amounts of GO, as shown in Figure 10. Quenching occurs due to the interaction between the molecule and GO. This was explored by studying the spectral behavior as a function of the chemical component ratio. By increasing the chemical ratio of PBD moiety in composites, the absorption spectra are broadened, and absorption increases. The fluorescence excitation spectra of the composites shown in Figure 10a, in contrast with those of PBD, show splitting of the bands into two sharp red-shifted bands when blending PBD with GO. This was explored by studying the spectral behavior as a function of the chemical component ratio of PBD and GO. From Figure 10a, it is seen that the fluorescence excitation spectrum intensity decreases with increasing GO content. Up to a chemical component ratio of 5:1 (w/w), the fluorescence excitation spectra show distinct splitting into two sets of bands. As small molecule, there exists the possibility of intermolecular aggregation for PBD. Figure 10b shows fluorescence emission spectra dependent on the chemical composition between GO and PBD. As the chemical component of PBD:GO changes from 10:1 to 2:1 (w/w), the maximum emission appears in a blue-shift and fluorescence intensity decreases when excited at 360 nm. The fluorescence intensity decreases with the incremental content of GO in composites. The fluorescent quenching effect of GO could be caused by GOs interaction with PBD. The fluorescence of the fluorescent derivatives could be quenched by trace of charged molecules (quenchers) via charge transfer or energy transfer [1]. The fluorescent small molecule would be absorbed on the surface of the GO sheets due to van der Waals forces between the nucleobases and the basal plane of the GO sheets. The absorption of PBD onto GO facilitates fluorescence quenching, since GO sheets act as a quencher (GO acceptor) that can absorb the energy from the fluorescent small molecule. The GO solution shows no detected fluorescence under various conditions. The added PBD was absorbed by the quencher GO, and quenched chromophore remained within the quenchers active sphere. Fluorescence quenching for systems of this type occurs intramolecularly.

### 3.4. Resistance-Switchable Mechanism

**Figure 11:**
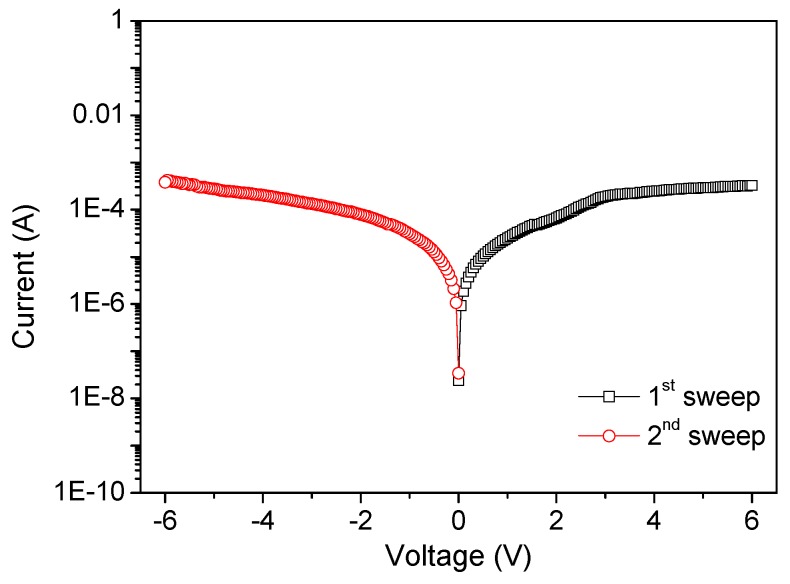
*I*-*V* characteristics of this control device.

To determine whether the resistance-switchable effect stems from the PBD:GO nanocomposite, a control experiment was conducted with GO as an active layer sandwiched between ITO and Ni electrodes. As exhibited in Figure 11, no hysteresis effects were observed in the *I*-*V* performance with single high resistivity. This result shows that the bistability and memory phenomena of the PBD:GO memory devices are derived from PBD:GO, which interestingly relies on the weight ratio between PBD and GO. *I*-*V* performance results illustrate that PBD attachment to the GO framework may help to tune the initial high resistivity [20,21].

**Figure 12:**
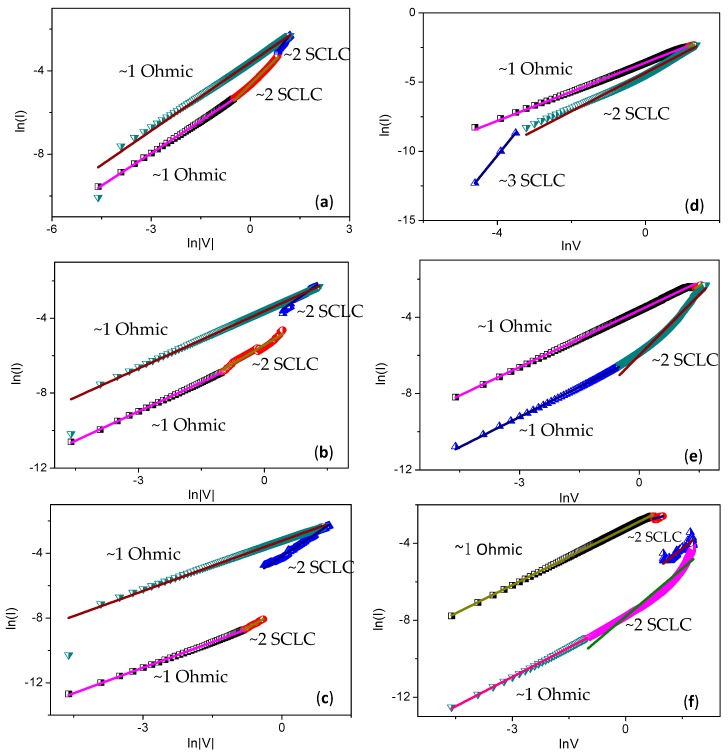
Measured and fitted *I*-*V* curves of the resistive switching memory devices based on PBD:GO blends. Measured and fitted semilogarithmic *I*-*V* curves for the component ratio of PBD:GO (**a**) 2:1, (**b**) 5:1, and (**c**) 10:1 (w/w) in negative sweeps; Plots of log*I*-log*V* with fitted conduction mechanism in positive sweeps for the component ratio of PBD:GO (**d**) 2:1, (**e**) 5:1, and (**f**) 10:1 (w/w).

The conduction mechanism of LRS and HRS in the PBD:GO nanocomposite layer can be explained by Ohmic and space-charge-limited conduction (SCLC) (Figure 12). To interpret the conduction mechanism, log*I*-log*V* plots were drawn for the write and erase processes. Fitting results suggest that SCLC is the conduction mechanism in the HRS region, whereas Ohmic conduction is dominant in the LRS region. In the write process, *I*-*V* characteristics in the HRS region consist of two distinct linear regions at low voltage, in which the relationship between current and voltage follows Ohm’s law (*I* ∝ *V*), the quantity of thermally generated free charge carriers exceeds that of injected carriers, and the curve is linear. In this region, traps are partially filled, and Ohmic behavior is observed. The region at high voltage conforms to the trap-filled limit current law (*I* ∝ *V*^2^). During transition voltage from Ohmic to SCLC, the electric field across the device is sufficient, so all traps are filled with charge carriers. In this quadratic region, the injected carriers are more dominant than thermally generated ones. The current, which is switchable from HRS to LRS, dramatically increases. When scanning again, the *I*-*V* curve is linear due to an Ohmic conduction mechanism. During the erase process, Ohmic conduction was also observed for LRS. The conduction from SCLC to Ohmic, after the current shifts from LRS to HRS, suddenly decreases. Therefore, the superior ReRAM properties of multilayers of GO blended with a 1,3,4-oxadiazole acceptor nanocomposite, according to the fitting results, are attributed to the electron traps caused by the strong electron acceptors [22].

## 4. Conclusions

In conclusion, we successfully demonstrated a nonvolatile organic memory using a small molecule absorbed in multilayers of GO (PBD:GO) as an active layer. In addition, this is the first successful attempt to fabricate an organic nonvolatile memory device using graphene-based small molecules with a cheap spin-coating technique. The resistance-switchable characteristics in a PBD:GO composite were studied by changing the chemical component ratio between PBD and GO. PBD:GO devices with a proportion 10:1 w/w exhibited better memory performance, and possessed a higher ON/OFF ratio (>10^2^) at a lower switching voltage of −0.67 V and a long retention ability. Investigation of a resistance-switchable mechanism was based on TEM, SEM, TGA and DTG, FTIR, Raman spectra, XRD, and fluorescence spectra. The superior ReRAM properties of multilayers of GO blended with PBD are attributed to the electron traps caused by the strong electron acceptors.
